# Bioprospecting Red Sea Coastal Ecosystems for Culturable Microorganisms and Their Antimicrobial Potential

**DOI:** 10.3390/md14090165

**Published:** 2016-09-10

**Authors:** Soha Al-Amoudi, Magbubah Essack, Marta F. Simões, Salim Bougouffa, Irina Soloviev, John A. C. Archer, Feras F. Lafi, Vladimir B. Bajic

**Affiliations:** 1 King Abdullah University of Science and Technology (KAUST), Computational Bioscience Research Center (CBRC), Thuwal 23955-6900, Saudi Arabia; soha.amoudi@kaust.edu.sa (S.A.-A.); magbubah.essack@kaust.edu.sa (M.E.); Simoesm@edgehill.ac.uk (M.F.S.); salim.bougouffa@kaust.edu.sa (S.B.); irinavsolo@gmail.com (I.S.); john.archer@kaust.edu.sa (J.A.C.A.); 2 Biology Department, Edge Hill University, St. Helens Road, Lancashire, Ormskirk L39 4QP, UK

**Keywords:** Red Sea, mangrove mud, microbial mat, barren soil, culturable bacteria, phylogenetic diversity, 16S rRNA, firmicutes, bioactivity, antimicrobial compounds, biosynthetic genes, bioinformatics

## Abstract

Microorganisms that inhabit unchartered unique soil such as in the highly saline and hot Red Sea lagoons on the Saudi Arabian coastline, represent untapped sources of potentially new bioactive compounds. In this study, a culture-dependent approach was applied to three types of sediments: mangrove mud (MN), microbial mat (MM), and barren soil (BS), collected from Rabigh harbor lagoon (RHL) and Al-Kharrar lagoon (AKL). The isolated bacteria were evaluated for their potential to produce bioactive compounds. The phylogenetic characterization of 251 bacterial isolates based on the 16S rRNA gene sequencing, supported their assignment to five different phyla: Proteobacteria, Firmicutes, Actinobacteria, Bacteroidetes, and Planctomycetes. Fifteen putative novel species were identified based on a 16S rRNA gene sequence similarity to other strain sequences in the NCBI database, being ≤98%. We demonstrate that 49 of the 251 isolates exhibit the potential to produce antimicrobial compounds. Additionally, at least one type of biosynthetic gene sequence, responsible for the synthesis of secondary metabolites, was recovered from 25 of the 49 isolates. Moreover, 10 of the isolates had a growth inhibition effect towards *Staphylococcus aureus*, *Salmonella typhimurium* and *Pseudomonas syringae.* We report the previously unknown antimicrobial activity of *B. borstelensis*, *P. dendritiformis* and *M. salipaludis* against all three indicator pathogens. Our study demonstrates the evidence of diverse cultured microbes associated with the Red Sea harbor/lagoon environments and their potential to produce antimicrobial compounds.

## 1. Introduction

The imprudent use of antibiotics to treat infectious diseases, along with their widespread use in agriculture and food manufacturing industries, has caused an increase in pathogen resistance which now poses a global public health threat associated with an increase in mortality rates and health care costs [1]. Unfortunately, the successes of screening soil actinomycetes for diffusible broad-spectrum antibiotic agents in the 1940s–1960s has met with diminishing returns, as in several instances, active compounds identified from screening assays have led to previously described compounds. Since the 1960s, only two new classes of antibiotics, the cyclic lipopeptide daptomycin, identified by a classical screening approach [2] and linezolid (a completely synthetic oxazolidinone) [3], have been successfully introduced to the clinic [4]. Since type-I polyketide synthases (PKS-I) and nonribosomal peptide synthetases (NRPS) have been found to support synthesis of secondary metabolites that act as antibiotics, immunosuppressants, toxins, siderophores, or antitumor agents, research is increasingly being focused on identifying microorganisms that harbor these multimodular enzymes [5] (see Table 1). The presence of these genes in the genome does not necessarily imply their expression or function, but does increase chances of identifying organisms capable of producing the above-mentioned bioactive compounds. Nonetheless, marine species that live in habitats with stressful conditions such as low light, low or high temperature, high pressure and salinity, produce different arrays of bioactive secondary metabolites that help them to survive [6], provide defense against other organisms, and help in the competition for food. These species represent an insufficiently explored source of organisms with potential to produce compounds high with antimicrobial activity.

Several recent studies have screened for antimicrobial compounds produced by microorganisms isolated from natural environments such as in hydrothermal vents [7], sediments [8], plants [9], seawater [10], and eukaryotic marine organisms [11]. This broader screening approach has successfully identified promising new antibiotics [12,13,14]. Research targeting sediments has sparked particular interest as soil compositions direct the diversity of the inhabiting microbial communities and their potential to produce antimicrobial compounds. Thus, microorganisms that inhabit unexplored unique soil compositions, such as in the Rabigh harbor lagoon (RHL) and Al-Kharrar lagoon (AKL) of the highly saline and hot Red Sea, represent untapped reservoirs of organisms that potentially produce novel bioactive compounds. RHL and AKL are located on the eastern coast of the Red Sea in Saudi Arabia. Previously, studies related to the sediment configuration of these lagoons have been reported [15,16,17,18]. Additionally, Red Sea mangrove metagenomics [19,20,21,22] have characterized biodiveristy or bioactivity as induced by Red Sea bacteria [11,23,24,25]. Nonetheless, both the lagoons have a higher nutrient content and organic matter when compared to the open sea, making their environments more favorable for this type of research. Additionally, RHL is considered a contaminated site owing to it being used as a harbor and being located near Petro Rabigh (Refining and Petrochemical Company), whereas AKL is considered less contaminated because its coast is poorly inhabited and only minor fishing activity is present in the lagoon.

In this study, we sampled sediments from three Red Sea environments: mangrove mud (MN), microbial mat (MM), and soil (BS) collected from the RHL and AKL. A culture-dependent approach was applied to these sediment samples for the isolation and identification of microorganisms using 16S rRNA gene sequencing. We have also reported their capacity to inhibit the growth of three laboratory pathogens, *Staphylococcus aureus* ATCC 25923, *Pseudomonas syringae* pv. *tomato* dc3000, and *Salmonella typhimurium* dT2, as well as their genomic potential to produce secondary metabolites with antimicrobial activity.

## 2. Results and Discussion

### 2.1. Bacterial Isolation

Characterizing microbial communities in sediment is a difficult task, mostly because of their huge genotypic and phenotypic diversity and heterogeneity [35]. In the top layer of soil, the bacterial inhabitants can be present in densities exceeding 10^9^ cells/g soil [35], and most of these strains seem to be unculturable. It is estimated that live culture techniques address 0.01%–1% of existing microbial diversity [20,36,37]. Only cultivated strains have been studied in detail [35,38], yet they represent less than 5% of the total microbial biomass in the soil [39]. Thus, in an attempt to broaden the diversity of the isolated strains, we employed several culture media: Difco marine agar 2216 (MA), 10% Difco marine agar 2216 (10% MA), Difco Marine agar 2216 with 1 g/L streptomycin (Anti-MA), actinomycetes isolation agar (AIA) and Difco marine broth 2216 gellan gum (MB-GM). Bacterial colonies grew fast on MA, exhibiting a more diverse colony morphology and density than on other media. Growth on the 10% MA medium tended to be similar to MA, but less diverse, while only a small number of colonies grew on Anti-MA and AIA. However, the growth in MB-GM was faster than in an agar-based media. This finding is not surprising as several studies have shown MB-GM to be more successful in isolating bacterial species from marine environments [40], and may isolate specifically rare actinomycetes [41,42]. Isolates were selected for propagation based on morphological distinctiveness (Supplementary Table S1).

### 2.2. 16S rRNA Gene Sequencing and Phylogenetic Analysis

**Figure 1:**
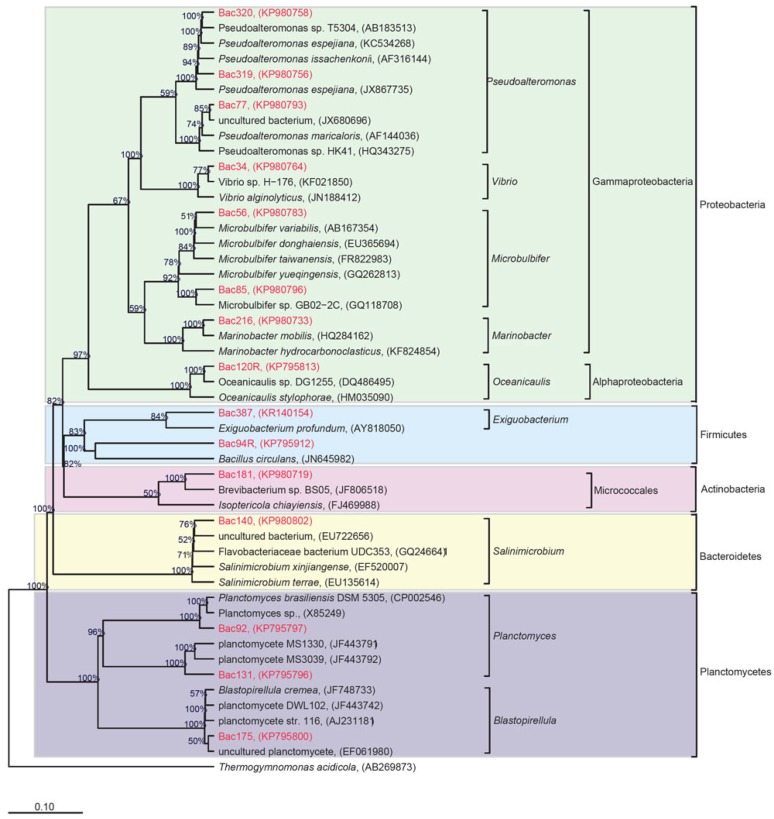
Phylogenetic tree showing the relationship of the 15 putative novel species. The sequence alignment was performed using the SINA online tool and trees were built in the software environment for sequence data called ARB, starting from the ribosomal RNA gene database called SILVA SSU dataset Ref NR 99 (version 119) using MrBayes with 100,000 Markov Chain Monte Carlo (mcmc) cycles.

**Figure 2:**
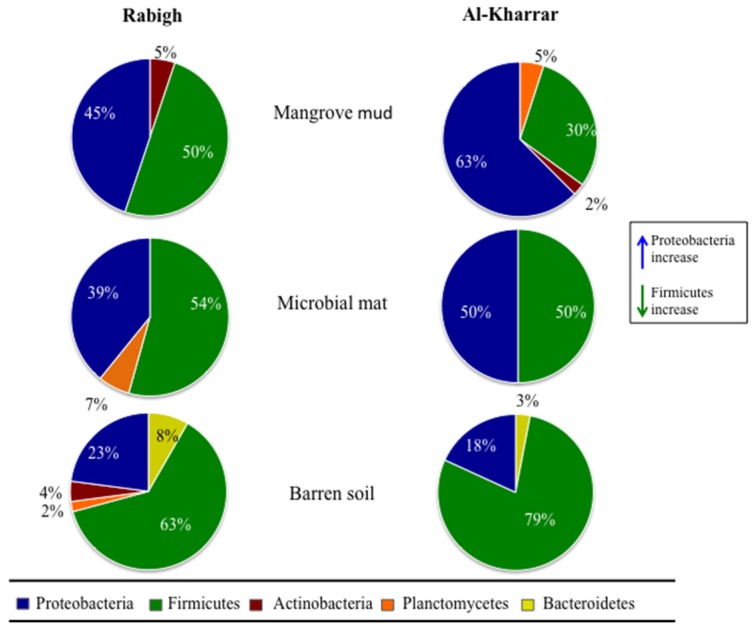
Overview of cultured microorganisms, at phylum level, isolated from Rabigh and Al-Kharrar sediments.

The 16S rRNA gene of the 251 isolated and purified bacterial strains, was sequenced as a taxonomic marker. Our analysis revealed that 16S rRNA gene sequences for all the strains showed similarity with 16S rRNA gene sequences deposited in the National Center for Biotechnology Information (NCBI) GenBank database in the range of 91%–100% (see Figure 1 and Supplementary Figure S1). The taxonomic classification placed the 251 isolates into five phyla comprising 32 genera: Firmicutes (135 strains; 12 genera), Proteobacteria (99 strains; 11 genera), Planctomycetes (six strains; two genera), Actinobacteria (six strains; four genera) and Bacteroidetes (five strains; three genera) (Figure 2). Firmicutes and Proteobacteria were isolated from all three types of sediments. Interestingly, the number of cultured Proteobacteria decreased in the three sediment types, which is in accordance with the reduction of the sediments exposure to seawater. In contrast, the number of cultured Firmicutes increased in the three sediment types, in accordance with the sediments exposure to seawater. The contrasting counts, for Proteobacteria and Firmicutes in these different sediments types, may be due to the spore-producing Firmicutes ability to survive in more adverse oligotrophic environments such as in BS compared to MN [43]. Additionally, Proteobacteria has the known ability to utilize chemical elements in soil more than other microorganisms, which makes the nutrient rich MN sediment more favorable for this phylum [44,45]. Bacteria from the Actinobacteria phylum were isolated from BS at RHL, and from MN collected from RHL and AKL, while Bacteroidetes were only isolated from BS collected from both locations. Planctomycetes were cultured from various sites such as MM and BS at RHL, and MN at AKL. The Firmicutes genus *Bacillus* was the predominant taxon isolated from all samples, especially from BS (Supplementary Table S1). The *Bacillus* species, recognized as an important source of natural bioactive products [46], are commonly found in nutrient-poor soils [47]. We found that the most abundant cultured and widely distributed species in RHL and AKL were: *Bacillus subtilis*, *Bacillus sonorensis* and *Bacillus licheniformis*. In addition, *Virgibacillus pantothenticus*, *Microbulbifer maritimus*, *Bacillus foraminis*, *Vibrio alginolyticus* and *Vibrio furnissii* strains were isolated three times or more from MN only.

Out of the 251 isolates, 15 could be considered putative novel species as they have ≤98% similarity to 16S rRNA gene sequences deposited in GenBank [48,49,50,51] (Table 2, Figure 1). The taxonomic classification placed the 15 isolates into five phyla comprising 12 genera and 15 species: Proteobacteria (*Vibrio alginolyticus*, *Microbulbifer maritimus*, *Microbulbifer gwangyangensis*, *Oceanicaulis* sp., *Marinobacter xestospongiae*, *Pseudoalteromonas espejiana*, *Pseudoalteromonas flavipulchra*, *Pseudoalteromonas atlantica*), Firmicutes (*Bacillus simplex* and *Exiguobacterium profundum*), Planctomycetes (*Planctomyces brasiliensis*, *Planctomycete* sp., *Blastopirellula cremea*), Actinobacteria (*Brevibacterium avium*) and Bacteroidetes (*Flavobacteriaceae bacterium*). Interestingly, 11 of the 15 putative novel species were isolated from the 10% MA medium. Thus, low nutrient media may be more suitable for targeting the isolation of those bacteria that are more difficult to culture—the rationale being that it provides an environment that does not facilitate the growth of easily culturable bacteria that thrive in high nutrient media and usually overshadow or outcompete the so-called “unculturable” bacteria. Moreover, from the putative novel species, 53.3% have a genetic affiliation with the phylum Proteobacteria, and 13.3% have a genetic affiliation with the phylum Firmicutes. Interestingly, 75.0% of putative novel strains were isolated from RHL.

### 2.3. Antimicrobial Screening

In order to defend themselves against other microorganisms, bacteria produce different compounds. Many of these metabolites are bioactive compounds and some have antimicrobial activity [52]. Within this context, we evaluated all 251 isolates for their antimicrobial activity against three pathogens: *Staphylococcus aureus* ATCC25923, *Pseudomonas syringae* dc3000, and *Salmonella typhimurium* dT2. The reason behind the selection of these pathogens was to cover the different spectrum of antimicrobial use. *S. aureus* causes several human infections such as skin and wound infections, septicemia, endocarditis, food poisoning, toxic shock syndrome, meningitis, pneumonia and osteomyelitis [53]. Additionally, the strain *S. aureus* ATCC 25923 is commonly used as a control to test for antibiotic susceptibility, and as a quality control strain for commercial products [54]. *P. syringae* pv. *tomato* dc3000 is a phytopathogen used as a model to study plant-bacterial interactions [55]. *S. enterica* serovar *typhimurium* definitive type 2 (DT2) is host restricted to *Columba livia* (rock or feral pigeon). However, it is also closely related to *S. typhimurium* isolates that circulate in livestock and causes zoonosis, that is, gastroenteritis in humans [56].

Of the 251 isolates screened, 49 isolates showed potential antimicrobial activity evidenced through growth inhibition against at least one of the indicator pathogens (included in Supplementary Table S3). These isolates belong to 12 genera of the phylum Firmicutes (genera: *Bacillus*, *Virgibacillus*, *Brevibacillus*, *Aneurinibacillus*, *Staphylococcus*, *Paenibacillus*), Proteobacteria (genera: *Microbulbifer*, *Salinivibrio*, *Pseudoalteromonas*, *Pseudomonas*, *Stenotrophomonas*) and Bacteroidetes (genus: *Pontibacter*). It is noteworthy that, of these 49 isolates, 67.0% were isolated from RHL and 48.9% have a genetic affiliation to genus *Bacillus*.

Of all isolates, 42 inhibited the growth of *S. aureus*, 14 isolates inhibited the growth of *S. typhimurium* and 34 isolates inhibited the growth of *P. syringae.* Only 10 isolates displayed zone inhibition against all of the three indicator laboratory pathogens (Table 3), of which nine isolates belong to the phylum Firmicutes, while one belongs to the phylum Proteobacteria (*Microbulbifer salipaludis* (Bac177)). Of these 10 isolates, only zone inhibition for *B. licheniformis* (Bac84) and *A. migulanus* (Bac271) showed an annular radius of ≥3.0 mm for all screenings, suggesting a higher activity against all three indicator laboratory pathogens. However, the strain Bac177 of *M. salipaludis* displayed the most effective zone inhibition against *S. typhimurium*. Two strains, Bac57 and Bac376, belonging to the species *Bacillus amyloliquefaciens* and *Virgibacillus olivae,* respectively, displayed strain-specific activity as shown by an annular radius of ≥3.0mm against *P. syringae.* Additionally, strains Bac389, Bac375 and Bac380 belonging to *Bacillus cereus*, *Pseudomonas fluorescens* and *Stenotrophomonas maltophilia* respectively, displayed strain-specific activity as shown by an annular radius of ≥3.0 mm against *S. aureus.* Of the putative novel strains, only a strain (Bac319) of *Pseudoalteromonas espejiana* displayed zone inhibition as shown by an annular radius of ≥1.0 mm against *S. aureus*, and a strain (Bac320) of *Pseudoalteromonas atlantica* revealed zone inhibition as shown by an annular radius of ≥1.0 mm against *S. aureus* and *P. syringae.*

Some of the ten strains that inhibited the growth of the three tested pathogens are already known to be producers of bioactive compounds. For example, Gramicidin S, a cyclodecapeptide recently isolated from *A. migulanus* [57], was originally isolated from the Gram-positive *Bacillus brevis* and was shown to exhibit an antibiotic effect against some Gram-positive and Gram-negative bacteria, as well as some fungi [58]. In addition, *B. licheniformis* is used for several biotechnological applications for its capacity to produce degrading enzymes such as proteases, lipases, pectate lyases, and polysaccharides [59]. *B. licheniformis* has further been shown to exhibit potent antimicrobial activity against indicator strains (*Lactococcus lactis*, *Lactobacillus bulgaricus* and *Listeria innocua*) and is clinically relevant (*Listeria monocytogenes*, *Staphylococcus aureus*, *Streptococcus agalactiae*, *Salmonella Typhimurium* and *Escherichia coli*) bacteria [60]. *B. subtilis* is a recognized producer of natural biocontrol agents, having high antifungal activity against *Alternaria solani*, *Botrytis cinerea*, *Monilia linhartiana* 869, *Phytophthora cryptogea* 759/1 and *Rhizoctonia* sp., and antibacterial activity against *Pseudomonas syringae* pv. tomato Ro and *Xanthomonas campestris* [61]. *B.* *sonorensis* has displayed antifungal activity against *Macrophomina phaseolina* [62] and has been shown to exhibit antibacterial activity against *Staphylococcus aureus* and *Listeria monocytogenes* as a potential food preservative [63]. *B. vallismortis* showed strong growth inhibition activity in vitro against the phytopathogens *Fusarium graminearum*, *Alternaria alternata*, *Rhizoctonia solani*, *Cryphonectria parasitica* and *Phytophthora capsici* [64]. So far, no specific antimicrobial activity has been detected for *B. borstelensis*, *P. dendritiformis* and *M. salipaludis*, which appears to be one of the novel findings of our study.

### 2.4. PCR-Screening for PKS and NRPS Domains

The 251 isolated strains were screened for the detection of genes involved in the synthesis of bioactive compounds: polyketide synthases type I (PKS-I) and type II (PKS-II), and nonribosomal peptide synthetases (NRPS) [65]. Although these enzymes are known to support synthesis of bioactive secondary metabolites such as antibiotics [66,67], their presence does not imply expression or functionality, but does increase the likelihood that the strain has the potential to produce antimicrobial compounds such as antibiotics, antitumor agents, or immunosuppressants. Only 107 of the isolated strains exhibited PCR products corresponding to at least one biosynthetic gene (see Supplementary Table S1).

For the 49 isolates that exhibited antimicrobial activity, at least one type of biosynthetic gene sequence was recovered from 25 strains. Of these 25 isolates, 56.0% were isolated from RHL, and 48.0% have a genetic affiliation to genus *Bacillus*. NRPS biosynthetic genes were identified in 40.8% of those selected strains, while PKS-I and PKS-II were identified in 28.5% and 20.4% of isolates respectively (Supplementary Table S3). Six isolates were positive for all biosynthetic genes screened: *B. licheniformis* (Bac84), *B. vallismortis* (Bac111), *P. espejiana* (Bac319), *B. subtilis* (Bac254) and *P. dendritiformis* (Bac363 and Bac390). For the isolates that displayed zone inhibition against all of the three indicator laboratory pathogens (Table 3), biosynthetic genes sequences were only found in five isolates: *B. licheniformis* (Bac84), *B. vallismortis* (Bac111), *B. subtilis* (Bac254), *P. dendritiformis* (Bac363 and Bac390). No biosynthetic gene sequences were recovered from the strains belonging to *B. borstelensis* (Bac98) *and M. salipaludis* (Bac177), bacteria for which antimicrobial activity has been reported in literature [68]. Additionally, from the 15 putative novel species (Table 2), biosynthetic gene sequences were recovered from nine strains belonging to the following species: *Microbulbifer gwangyangensis* (Bac85), Planctomyces brasiliensis (Bac 92), Bacillus simplex (Bac 94), *Blastopirellula cremea* (Bac175), *Brevibacterium avium* (Bac181), *Marinobacter xestospongiae* (Bac216), *P. atlantica* (Bac288), *P. espejiana* (Bac319) and *Exiguobacterium profundum* (Bac387).

Our study shows a rich biodiversity of culturable Firmicutes (Supplementary Table S2) from Red Sea ecosystems on the Saudi Arabian coastline. Of all isolated strains, we found that 49 exhibit potential antimicrobial activity. From these, 25 have at least one type of biosynthetic gene sequence, indicating that these isolates are a valuable resource for the potential discovery of bioactive compounds. Moreover, 75% of putative novel species and 67% of strains that exhibited antimicrobial activity were isolated from RHL.

In this process we have also managed to short list 15 putative novel species that require further experimentation to verify species novelty such as whole genome sequencing. In addition, five additional strains (Bac 84, 111, 254, 363, 390) will be shortlisted for the purification and identification of their antimicrobial compounds using High Performance Liquid Chromotography (HPLC), Nuclear Magnetic Resonance (NMR) and Mass Spectrometry (MS).

## 3. Material and Methods

### 3.1. Sediment Sampling

**Figure 3:**
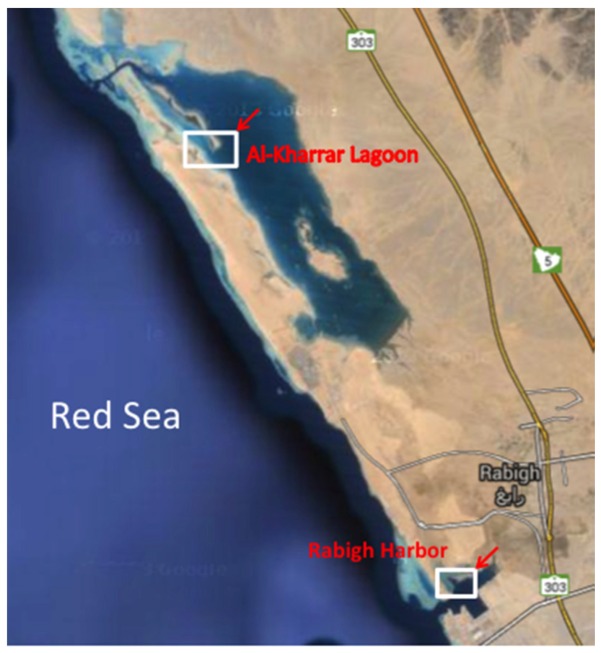
Map showing the sampling locations.

Three types of sediment samples: mangrove mud (MN), microbial mat (MM), and barren soil (BS) were collected from RHL (39°0′35.762′′ E, 22°45′5.582′′ N) and AKL (38°54′39.638′′ E, 22°54′50.251′′ N) in Saudi Arabia (Figure 3). MN mud was sampled from the coastal lagoon of the Red Sea alongside mangrove tree. MM was sampled approximately 7.5 m away from the coastal lagoon, while BS represents sediment that did not come in contact with the lagoon water at high tide. All samples were collected in triplicate from the same depth (2–25 cm), using a hollow steel tube (core sampler—each core was 20–25 cm in length and 5 cm in diameter). Cores were kept on ice until the processing of samples were done on the same day. Eight grams of wet weight sediment were homogenized at low speed with 10 mL of sterilised Red Sea water. The supernatant (100 μL) was diluted five- and 25-fold, and subsequently plated out on different media. Processing was the same for all samples.

### 3.2. Isolation and Purification of Bacterial Strains

Several selective media types were used: actinomycetes isolation agar for cultivating Actinobacteria, Difco Marine agar 2216 for cultivating heterotrophic bacteria, 10% Difco Marine agar 2216 for cultivating oligotrophic bacteria, Difco Marine agar 2216 with 1 g/L streptomycin for bacteria that are resistant to antibiotic, and Difco Marine broth 2216 gellan gum media for bacteria that do not prefer agar (BD Difco, Franklin Lakes, NJ, USA). All media were prepared in artificial seawater. Inoculated plates were incubated at 28 °C for up to 28 days and colonies were selected based on morphology. After successive transfers, pure isolates were frozen at −80 °C in ddH_2_O (for DNA extraction and amplification) and 30% glycerol solution for long-term storage.

### 3.3. Molecular Identification and Phylogenetic Analysis

Genomic DNA was prepared as described previously [69], but with slight modification of the phenol-chloroform extraction and ethanol precipitation, which were done according to [70]. For 16S rRNA gene amplification, universal primers 27F primer (5′-AGAGTTTGATCMTGGCTCAG-3′) and 1492R primer (5′-GGTTACCTTGTTACGACTT-3′), were used in the PCR reaction as previously described [71], with some parameters modified to the thermocycler (Bio-Rad, Hercules, CA, USA) used. Initial denaturation occurred at 95 °C for 7 min, followed by 35 cycles of: denaturation at 95 °C for 1 min, annealing at 54 °C for 45 s, and an extension at 72 °C for 90 min, with a final extension at 72 °C for 5 min. Amplified DNA was electrophoresed on 1% agarose gel. The amplified PCR products of bacterial gene fragments were purified according to the manufacturers recommendations: Nucleospin recommended protocol (Macherey-Nagel GmbH, Düren, North Rhine-Westphalia, Germany). Purified products were submitted for Sanger sequencing of the 16S rRNA gene at the King Abdullah University of Science and Technology (KAUST) Bioscience Core Laboratory. The 16S rRNA gene sequences were compared to sequences within the NCBI database using the Basic Local Alignment Search Tool (BLAST). The phylogenetic tree was built in the ARB package [72], using the SILVA SSU Ref NR 99 (version 119) as the reference tree [73]. Alignment of the 16s rRNA genes (also referred to as species in the text) was performed using SILVA’s SINA on-line aligner [74], with the variability profile set to “Bacteria”, and all other parameters set to default. The alignment was inspected and edited in the ARB package. Initially, we added our species to the reference tree using the parsimony method. Close relatives of the reference tree were selected and a new tree was built using MrBayes [75] in ARB (filter: bacteria, number of substitution type 4, number of cycles for Markov Chain Monte Carlo = 100,000, number of chains = 6, temperature parameter for heating the chains = 0.5, Markov chain sample frequency = 500). *Thermogymnomonas acidicola* (archaea) was added to the tree as an outgroup. The 16S rRNA gene sequences of the isolates were deposited in GenBank under the following accession numbers KP795796-KP795924 and KP980708-KP980808.

### 3.4. Screening for Antimicrobial Activity

**Figure 4:**
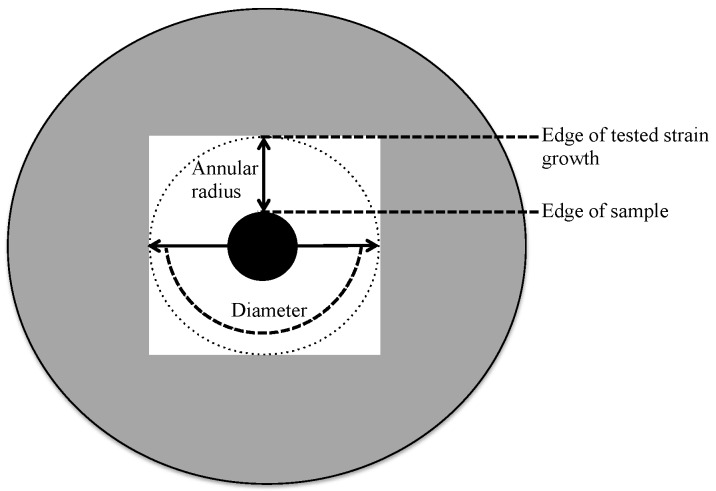
Sketch depicting the annular radius and diameter of the zone of inhibition.

Three pathogenic test laboratory strains (*Staphylococcus aureus* strain ATCC25923, *Pseudomonas syringae* strain dc3000 and *Salmonella typhimurium* strain dT2) were used to analyze their sensitivity towards the isolated stains. LB agar (Fisher BioReagents, Miller, Pittsburgh, PA, USA) was prepared and autoclaved at 121 °C for 20 min. Stock cultures of the three pathogenic strains and all isolates were cultured individually in LB agar plates and then incubated overnight at 37 °C. One of the grown colonies was collected and mixed individually with 1 mL of LB broth in a 1.5 μL tube and incubated at 37 °C, with shaking at 250 rpm overnight. A new LB agar plate was spread with a 100 μL aliquot of an individual pathogenic strain. Next, the 10 μL aliquot of isolate suspension (optical density 600 nm (OD_600_) of 0.5) was applied on the inoculated plate which was then incubated overnight aerobically at 37 °C. The inhibition zone of the annular radius and diameter was then been registered (Figure 4) after 24 h.

### 3.5. PCR Screening for PKS and NRPS Gene Fragments

Specific primers used to screen for domains associated with the PKS and NRPS genes, are listed in Table 4 [65,76]. The PCR products were electrophoresed on a 1% agarose gel, from which the bands of interest (NRPS: 700–800 bp; PKS-I: 1250–1400 bp; PKS-II: 800–900 bp) were cut out and purified using the Wizard^®^ SV Gel and PCR Clean-Up System (Promega, Madison, WI, USA). PCR products were cloned using pGEM^®^-T and pGEM^®^-T Easy vectors (Promega, Madison, WI, USA). Plasmids that contain the PCR products were purified using the QIAprep^®^ Spin Miniprep kit (Qiagen, Hilden, North Rhine-Westphalia, Germany). PCR products were then sequenced and compared with PKS and NRPS sequences in the NCBI database by using the Basic Local Alignment Search Tool X (BLASTX).

## 4. Conclusions

Our study shows a rich biodiversity of culturable Firmicutes (Supplementary Table S2) from Red Sea ecosystems on the Saudi Arabian coastline. Of all isolated strains, we found that 49 exhibit potential antimicrobial activity. From these, 25 have at least one type of biosynthetic gene sequence, indicating that these isolates are a valuable resource for the potential discovery of bioactive compounds. Moreover, 75% of putative novel species and 67% of strains that exhibited antimicrobial activity were isolated from RHL.

In this process, we have also managed to short list 15 putative novel species that require further experimentation to verify species novelty such as whole genome sequencing. In addition, five additional strains (Bac 84, 111, 254, 363, 390) will be shortlisted for the purification and identification of their antimicrobial compounds using High Performance Liquid Chromotography (HPLC), Nuclear Magnetic Resonance (NMR) and Mass Spectrometry (MS).

## Figures and Tables

**Table 1:** Nonribosomal peptide synthetases (NRPS) and polyketide synthases (PKS) compounds derived from microbes.

Compound	Enzyme	Source	Activity	Reference
Bacitracin	NRPS	*Bacillus* spp.	Antibacterial	[26]
Surfactin	NRPS	*Bacillus subtilis*	Antibacterial	[27]
Macrolactin	PKS	*Bacillus amyloliquefaciens*	Antibacterial	[28]
Mupirocin	PKS	*Pseudomonads fluorescens*	Antibacterial	[29]
Retimycin	NRPS	*Sallinispora arenicola*	Antitumor	[30]
Pederin	PKS	*Paederus fuscipes*	Antitumor	[31]
Salinosporamide K	NRPS	*Salinispora pacifica*	Antitumor	[32]
Salinilactam A	PKS	*Salinispora tropica*	Antitumor	[33]
Bryostatin	PKS	*Candidatus* *Endobugula sertula*	Antitumor	[34]

**Table 2:** 16S rRNA gene taxonomic affiliation of the putative novel isolated strains.

Strain ID	% Similarity of 16S rRNA Sequences to GenBank Strains:	Number of Nucleotides (bp)	Phylum	Location	Sediment Type	Media
Bac34	98% *Vibrio alginolyticus*	1429	Proteobacteria	RHL	MN	10% MA
Bac56	98% *Microbulbifer maritimus*	1390	Proteobacteria	RHL	MN	10% MA
Bac77	98% *Pseudoalteromonas flavipulchra*	1406	Proteobacteria	RHL	MM	10% MA
Bac85	95% *Microbulbifer gwangyangensis*	1406	Proteobacteria	RHL	MM	10% MA
Bac92	97% *Planctomyces brasiliensis*	1372	Planctomycete	RHL	MM	Anti-MA
Bac94	98% *Bacillus simplex*	1423	Firmicutes	RHL	MM	10% MA
Bac120	98% *Oceanicaulis* sp.	1336	Proteobacteria	RHL	BS	10% MA
Bac131	98% *Planctomycete* sp.	1417	Planctomycete	RHL	BS	Anti-MA
Bac140	97% *Flavobacteriaceae bacterium*	1394	Bacteroidetes	RHL	BS	10% MA
Bac175	98% *Blastopirellula cremea*	1385	Planctomycete	AKL	MN	Anti-MA
Bac181	91% *Brevibacterium avium*	1341	Actinobacteria	AKL	MN	AIA
Bac216	97% *Marinobacter xestospongiae*	1414	Proteobacteria	AKL	MM	10% MA
Bac319	98% *Pseudoalteromonas espejiana*	1380	Proteobacteria	RHL	MM	10% MA
Bac320	98% *Pseudoalteromonas atlantica*	1392	Proteobacteria	RHL	MM	MB-GM
Bac387	96% *Exiguobacterium profundum*	1327	Firmicutes	RHL	MN	10% MA

Abbreviations: RHL: Rabigh harbor lagoon; AKL: Al-Kharrar lagoon MN: mangrove mud; MM: microbial mat; BS: barren soil; 10% MA: 10% Difco Marine agar 2216; Anti-MA: Difco Marine agar 2216 with 1 g/L streptomycin; AIA: actinomycetes isolation agar; MB-GM: Difco Marine broth 2216 gellan gum media.

**Table 3:** Isolates that displayed zone inhibition against all of the three indicator laboratory pathogens.

Closest Phylogenetic Relative by BLAST	Sequence Similarity (%)	Zone of Inhibition (mm)					
		*Staphylococcus aureus*		*Salmonella typhimurium*		*Pseudomonas syringae*	
		Annular radius	Diameter	Annular radius	Diameter	Annular radius	Diameter
*Bacillus licheniformis*	99	4.5	14	3	11	3	11
*Bacillus sonorensis*	99	3.8	12	2.8	10	3	11
*Brevibacillus borstelensis*	99	5	15	0.5	6	4.3	13.5
*Bacillus vallismortis*	99	2	9	4	13	0.5	6
*Microbulbifer salipaludis*	99	3.3	11	6.5	18	1	7
*Bacillus subtilis*	99	5	15	3	11	0.5	6
*Aneurinibacillus migulanus*	99	4	13	2	9	3.3	11.5
*Aneurinibacillus migulanus*	99	5.5	16	3	11	4.5	14
*Paenibacillus dendritiformis*	99	0.5	6	3	11	1	7
*Paenibacillus dendritiformis*	99	2	9	2	6	5	15

**Table 4:** Primers used to screen for domains associated with the PKS and NRPS genes.

Primer	Sequence	Target	Reference
A7R	SASGTCVCCSGTSCGGTAS	NRPS	[65]
A3	GCSTACSYSATSTACACSTCSGG		
K1	TSAAGTCSAACATCGGBCA	PKS-I	[65]
M6R	CGCAGGTTSCSGTACCAGTA		
KSα	TSGRCTACRTCAACGGSCACGG	PKS-II	[77]
KSβ	TACSAGTCSWTCGCCTGGTTC		

## Supplementary Materials

The following are available online at www.mdpi.com/1660-3397/14/9/165/s1.
