# Supplementary material for: Bioprospecting Red Sea Coastal Ecosystems for Culturable Microorganisms and Their Antimicrobial Potential
Source: Mar Drugs. 2016 Sep 10;14(9):165. doi: 10.3390/md14090165 (PMC5039536; doi:10.3390/md14090165)
Supplement: Supplementary file 1 [file marinedrugs-14-00165-s001.zip › marinedrugs-14-00165/Supplementary Figure S1.pdf]

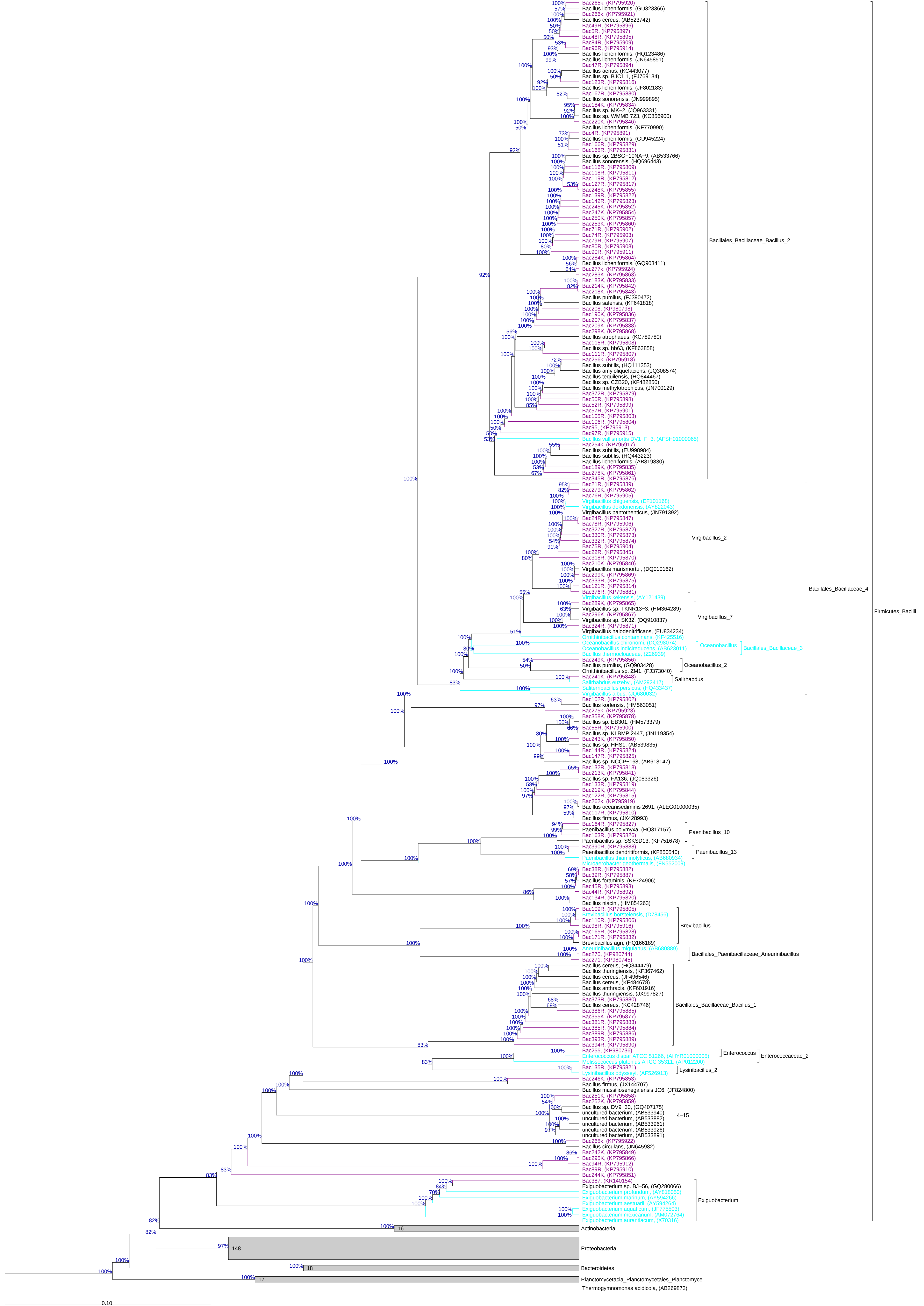
Bac265k, (KP795920)
Bacillus licheniformis, (GU323366)
Bac266K, (KP795921)
Bacillus cereus, (AB523742)
Bac49R, (KP795896)
Bac5R, (KP795897)
Bac48R, (KP795895)
Bac84R, (KP795909)
Bac96R, (KP795914)
Bacillus licheniformis, (HQ123486)
Bacillus licheniformis, (JN645851)
Bac47R, (KP795894)
Bacillus aerius, (KC443077)
Bacillus sp. BJC1.1, (FJ769134)
Bac123R, (KP795816)
Bacillus licheniformis, (JF802183)
Bac167R, (KP795830)
Bacillus sonorensis, (JN999895)
Bac184K, (KP795834)
Bacillus sp. MK−2, (JQ963331)
Bacillus sp. WMMB 723, (KC856900)
Bac220K, (KP795846)
Bacillus licheniformis, (KF770990)
Bac4R, (KP795891)
Bacillus licheniformis, (GU945224)
Bac166R, (KP795829)
Bac168R, (KP795831)
Bacillus sp. 2BSG−10NA−9, (AB533766)
Bacillus sonorensis, (HQ696443)
Bac116R, (KP795809)
Bac118R, (KP795811)
Bac119R, (KP795812)
Bac127R, (KP795817)
Bac248K, (KP795855)
Bac139R, (KP795822)
Bac142R, (KP795823)
Bac245K, (KP795852)
Bac247K, (KP795854)
Bac250K, (KP795857)
Bac253K, (KP795860)
Bac71R, (KP795902)
Bac74R, (KP795903)
Bac79R, (KP795907)
Bac80R, (KP795908)
Bac90R, (KP795911)
Bac284K, (KP795864)
Bacillus licheniformis, (GQ903411)
Bac277k, (KP795924)
Bac283K, (KP795863)
Bac183K, (KP795833)
Bac214K, (KP795842)
Bac218K, (KP795843)
Bacillus pumilus, (FJ390472)
Bacillus safensis, (KF641818)
Bac208, (KP980798)
Bac190K, (KP795836)
Bac207K, (KP795837)
Bac209K, (KP795838)
Bac298K, (KP795868)
Bacillus atrophaeus, (KC789780)
Bac115R, (KP795808)
Bacillus sp. hb63, (KF863858)
Bac111R, (KP795807)
Bac256k, (KP795918)
Bacillus subtilis, (HQ111353)
Bacillus amyloliquefaciens, (JQ308574)
Bacillus tequilensis, (HQ844467)
Bacillus sp. CZB20, (KF482850)
Bacillus methylotrophicus, (JN700129)
Bac372R, (KP795879)
Bac50R, (KP795898)
Bac52R, (KP795899)
Bac57R, (KP795901)
Bac105R, (KP795803)
Bac106R, (KP795804)
Bac95, (KP795913)
Bac97R, (KP795915)
Bacillus vallismortis DV1−F−3, (AFSH01000065)
Bac254k, (KP795917)
Bacillus subtilis, (EU998984)
Bacillus subtilis, (HQ443223)
Bacillus licheniformis, (AB819830)
Bac189K, (KP795835)
Bac278K, (KP795861)
Bac345R, (KP795876)
Bacillales_Bacillaceae_Bacillus_2
Bac21R, (KP795839)
Bac279K, (KP795862)
Bac76R, (KP795905)
Virgibacillus chiguensis, (EF101168)
Virgibacillus dokdonensis, (AY822043)
Virgibacillus pantothenticus, (JN791392)
Bac24R, (KP795847)
Bac78R, (KP795906)
Bac327R, (KP795872)
Bac330R, (KP795873)
Bac332R, (KP795874)
Bac75R, (KP795904)
Bac22R, (KP795845)
Bac318R, (KP795870)
Bac210K, (KP795840)
Virgibacillus marismortui, (DQ010162)
Bac299K, (KP795869)
Bac333R, (KP795875)
Bac121R, (KP795814)
Bac376R, (KP795881)
Virgibacillus_2
Virgibacillus kekensis, (AY121430)
Bac289K, (KP795865)
Virgibacillus sp. TKNR13−3, (HM364289)
Bac296K, (KP795867)
Virgibacillus sp. SK32, (DQ910837)
Bac324R, (KP795871)
Virgibacillus halodenitrificans, (EU834234)
Virgibacillus_7
Ornithinibacillus contaminans, (KF425516)
Oceanobacillus chironomi, (DQ298074)
Oceanobacillus indicireducens, (AB623011)
Bacillus thermocloaceae, (Z26939)
Oceanobacillus
Bacillales_Bacillaceae_3
Bac249K, (KP795856)
Bacillus pumilus, (GQ903428)
Ornithinibacillus sp. ZM1, (FJ373040)
Oceanobacillus_2
Bac241K, (KP795848)
Salirhabdus euzebyi, (AM292417)
Salirhabdus
Saliterribacillus persicus, (HQ433437)
Virgibacillus albus, (JQ680032)
Bacillales_Bacillaceae_4
Bac102R, (KP795802)
Bacillus korlensis, (HM563051)
Bac275k, (KP795923)
Bac358K, (KP795878)
Bacillus sp. EB301, (HM573379)
Bac55R, (KP795900)
Bacillus sp. KLBMP 2447, (JN119354)
Bac243K, (KP795850)
Bacillus sp. HHS1, (AB539835)
Bac144R, (KP795824)
Bac147R, (KP795825)
Bacillus sp. NCCP−168, (AB618147)
Bac132R, (KP795818)
Bac213K, (KP795841)
Bacillus sp. FA136, (JQ083326)
Bac133R, (KP795819)
Bac219K, (KP795844)
Bac122R, (KP795815)
Bac262k, (KP795919)
Bacillus oceanisediminis 2691, (ALEG01000035)
Bac117R, (KP795810)
Bacillus firmus, (JX428993)
Bac164R, (KP795827)
Paenibacillus polymyxa, (HQ317157)
Bac163R, (KP795826)
Paenibacillus sp. SSKSD13, (KF751678)
Paenibacillus_10
Bac390R, (KP795888)
Paenibacillus dendritiformis, (KF850540)
Paenibacillus thiaminolyticus, (AB680934)
Paenibacillus_13
Microaerobacter geothermalis, (FN552009)
Bac38R, (KP795882)
Bac39R, (KP795887)
Bacillus foraminis, (KF724906)
Bac45R, (KP795893)
Bac44R, (KP795892)
Bac134R, (KP795820)
Bacillus niacini, (HM854263)
Bac109R, (KP795805)
Brevibacillus borstelensis, (D78456)
Bac110R, (KP795806)
Bac98R, (KP795916)
Bac165R, (KP795828)
Bac171R, (KP795832)
Brevibacillus agri, (HQ166189)
Brevibacillus
Aneurinibacillus migulanus, (AB680889)
Bac270, (KP980744)
Bac271, (KP980745)
Bacillales_Paenibacillaceae_Aneurinibacillus
Bacillus cereus, (HQ844479)
Bacillus thuringiensis, (KF367462)
Bacillus cereus, (JF496546)
Bacillus cereus, (KF484678)
Bacillus anthracis, (KF601916)
Bacillus thuringiensis, (JX997827)
Bac373R, (KP795880)
Bacillus cereus, (KC428746)
Bac386R, (KP795885)
Bac355K, (KP795877)
Bac381R, (KP795883)
Bac385R, (KP795884)
Bac389R, (KP795886)
Bac393R, (KP795889)
Bac394R, (KP795890)
Bacillales_Bacillaceae_Bacillus_1
Bac255, (KP980736)
Enterococcus dispar ATCC 51266, (AHYR01000005)
Melissococcus plutonius ATCC 35311, (AP012200)
Enterococcus
Enterococcaceae_2
Bac135R, (KP795821)
Lysinibacillus odysseyi, (AF526913)
Lysinibacillus_2
Bac246K, (KP795853)
Bacillus firmus, (JX144707)
Bacillus massiliosenegalensis JC6, (JF824800)
Bac251K, (KP795858)
Bac252K, (KP795859)
Bacillus sp. DV9−30, (GQ407175)
uncultured bacterium, (AB533940)
uncultured bacterium, (AB533882)
uncultured bacterium, (AB533961)
uncultured bacterium, (AB533926)
uncultured bacterium, (AB533891)
4−15
Bac268k, (KP795922)
Bacillus circulans, (JN645982)
Bac242K, (KP795849)
Bac295K, (KP795866)
Bac94R, (KP795912)
Bac89R, (KP795910)
Bac244K, (KP795851)
Bac387, (KR140154)
Exiguobacterium sp. BJ−56, (GQ280066)
Exiguobacterium profundum, (AY818050)
Exiguobacterium marinum, (AY594266)
Exiguobacterium aestuarii, (AY594264)
Exiguobacterium aquaticum, (JF775503)
Exiguobacterium mexicanum, (AM072764)
Exiguobacterium aurantiacum, (X70316)
Exiguobacterium
Firmicutes_Bacilli
16
Actinobacteria
148
Proteobacteria
18
Bacteroidetes
17
Planctomycetacia_Planctomycetales_Planctomyce
Thermogymnomonas acidicola, (AB269873)
0.10

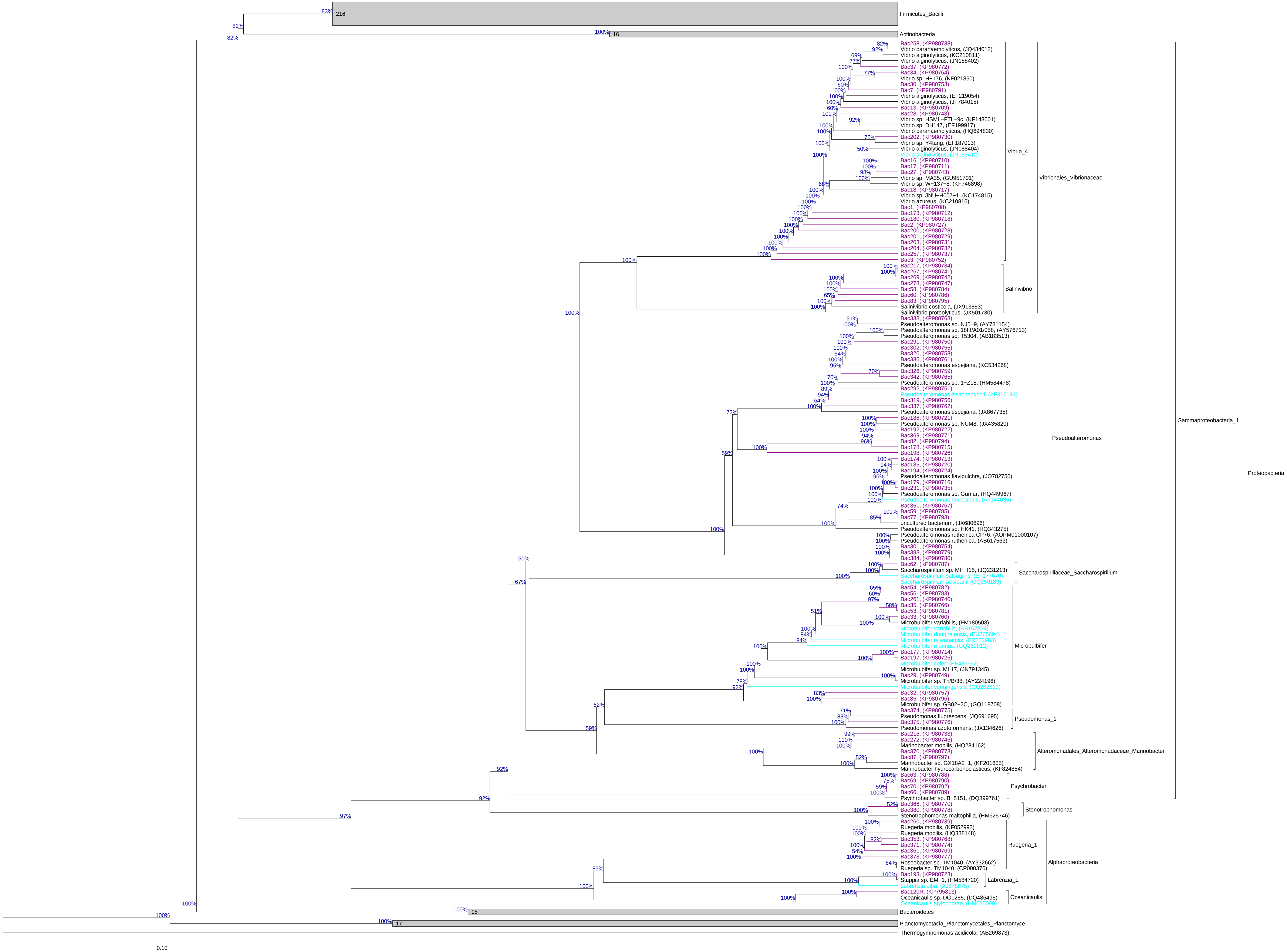

Firmicutes_Bacilli
Actinobacteria
Bac258, (KP980738)
Vibrio parahaemolyticus, (JQ434012)
Vibrio alginolyticus, (KC210811)
Vibrio alginolyticus, (JN188402)
Bac37, (KP980772)
Bac34, (KP980764)
Vibrio sp. H−176, (KF021850)
Bac30, (KP980753)
Bac7, (KP980791)
Vibrio alginolyticus, (EF219054)
Vibrio alginolyticus, (JF784015)
Bac13, (KP980709)
Bac28, (KP980748)
Vibrio sp. HSML−FTL−9c, (KF148601)
Vibrio sp. DH147, (EF199917)
Vibrio parahaemolyticus, (HQ694830)
Bac202, (KP980730)
Vibrio sp. Y4tang, (EF187013)
Vibrio alginolyticus, (JN188404)
Vibrio alginolyticus, (JN188412)
Bac16, (KP980710)
Bac17, (KP980711)
Bac27, (KP980743)
Vibrio sp. MA35, (GU951701)
Vibrio sp. W−137−8, (KF746898)
Bac18, (KP980717)
Vibrio sp. JNU−H007−1, (KC174815)
Vibrio azureus, (KC210816)
Bac1, (KP980708)
Bac173, (KP980712)
Bac180, (KP980718)
Bac2, (KP980727)
Bac200, (KP980728)
Bac201, (KP980729)
Bac203, (KP980731)
Bac204, (KP980732)
Bac257, (KP980737)
Bac3, (KP980752)
Bac217, (KP980734)
Bac267, (KP980741)
Bac269, (KP980742)
Bac273, (KP980747)
Bac58, (KP980784)
Bac60, (KP980786)
Bac83, (KP980795)
Salinivibrio costicola, (JX913853)
Salinivibrio proteolyticus, (JX501730)
Bac338, (KP980763)
Pseudoalteromonas sp. NJ5−9, (AY781154)
Pseudoalteromonas sp. 18III/A01/058, (AY576713)
Pseudoalteromonas sp. T5304, (AB183513)
Bac291, (KP980750)
Bac302, (KP980755)
Bac320, (KP980758)
Bac336, (KP980761)
Pseudoalteromonas espejiana, (KC534268)
Bac326, (KP980759)
Bac342, (KP980765)
Pseudoalteromonas sp. 1−Z18, (HM584478)
Bac292, (KP980751)
Pseudoalteromonas issachenkonii, (AF316144)
Bac319, (KP980756)
Bac337, (KP980762)
Pseudoalteromonas espejiana, (JX867735)
Bac186, (KP980721)
Pseudoalteromonas sp. NUM8, (JX435820)
Bac192, (KP980722)
Bac369, (KP980771)
Bac82, (KP980794)
Bac178, (KP980715)
Bac198, (KP980726)
Bac174, (KP980713)
Bac185, (KP980720)
Bac194, (KP980724)
Pseudoalteromonas flavipulchra, (JQ782750)
Bac179, (KP980716)
Bac231, (KP980735)
Pseudoalteromonas sp. Gumar, (HQ449967)
Pseudoalteromonas maricaloris, (AF144036)
Bac351, (KP980767)
Bac59, (KP980785)
Bac77, (KP980793)
uncultured bacterium, (JX680696)
Pseudoalteromonas sp. HK41, (HQ343275)
Pseudoalteromonas ruthenica CP76, (AOPM01000107)
Pseudoalteromonas ruthenica, (AB617563)
Bac301, (KP980754)
Bac383, (KP980779)
Bac384, (KP980780)
Bac62, (KP980787)
Saccharospirillum sp. MH−I15, (JQ231213)
Saccharospirillum salsuginis, (EF177670)
Saccharospirillum aestuarii, (GQ250189)
Bac54, (KP980782)
Bac56, (KP980783)
Bac261, (KP980740)
Bac35, (KP980766)
Bac53, (KP980781)
Bac33, (KP980760)
Microbulbifer variabilis, (FM180508)
Microbulbifer variabilis, (AB167354)
Microbulbifer donghaiensis, (EU365694)
Microbulbifer taiwanensis, (FR822983)
Microbulbifer marinus, (GQ262812)
Bac177, (KP980714)
Bac197, (KP980725)
Microbulbifer celer, (EF486352)
Microbulbifer sp. ML17, (JN791345)
Bac29, (KP980749)
Microbulbifer sp. Th/B/38, (AY224196)
Microbulbifer yueqingensis, (GQ262813)
Bac32, (KP980757)
Bac85, (KP980796)
Microbulbifer sp. GB02−2C, (GQ118708)
Bac374, (KP980775)
Pseudomonas fluorescens, (JQ691695)
Bac375, (KP980776)
Pseudomonas azotoformans, (JX134626)
Bac216, (KP980733)
Bac272, (KP980746)
Marinobacter mobilis, (HQ284162)
Bac370, (KP980773)
Bac87, (KP980797)
Marinobacter sp. GX18A2−1, (KF201605)
Marinobacter hydrocarbonoclasticus, (KF824854)
Bac63, (KP980788)
Bac69, (KP980790)
Bac70, (KP980792)
Bac66, (KP980789)
Psychrobacter sp. B−5151, (DQ399761)
Bac366, (KP980770)
Bac380, (KP980778)
Stenotrophomonas maltophilia, (HM625746)
Bac260, (KP980739)
Ruegeria mobilis, (KF052993)
Ruegeria mobilis, (HQ338148)
Bac353, (KP980768)
Bac371, (KP980774)
Bac361, (KP980769)
Bac378, (KP980777)
Roseobacter sp. TM1040, (AY332662)
Ruegeria sp. TM1040, (CP000376)
Bac193, (KP980723)
Stappia sp. EM−1, (HM584720)
Labrenzia alba, (AJ878875)
Bac120R, (KP795813)
Oceanicaulis sp. DG1255, (DQ486495)
Oceanicaulis stylophorae, (HM035090)
Bacteroidetes
Planctomycetacia_Planctomycetales_Planctomyce
Thermogymnomonas acidicola, (AB269873)
Vibrio_4
Salinivibrio
Vibrionales_Vibrionaceae
Pseudoalteromonas
Saccharospirillaceae_Saccharospirillum
Microbulbifer
Pseudomonas_1
Alteromonadales_Alteromonadaceae_Marinobacter
Psychrobacter
Stenotrophomonas
Ruegeria_1
Labrenzia_1
Oceanicaulis
Alphaproteobacteria
Gammaproteobacteria_1
Proteobacteria
216
16
18
17
0.10

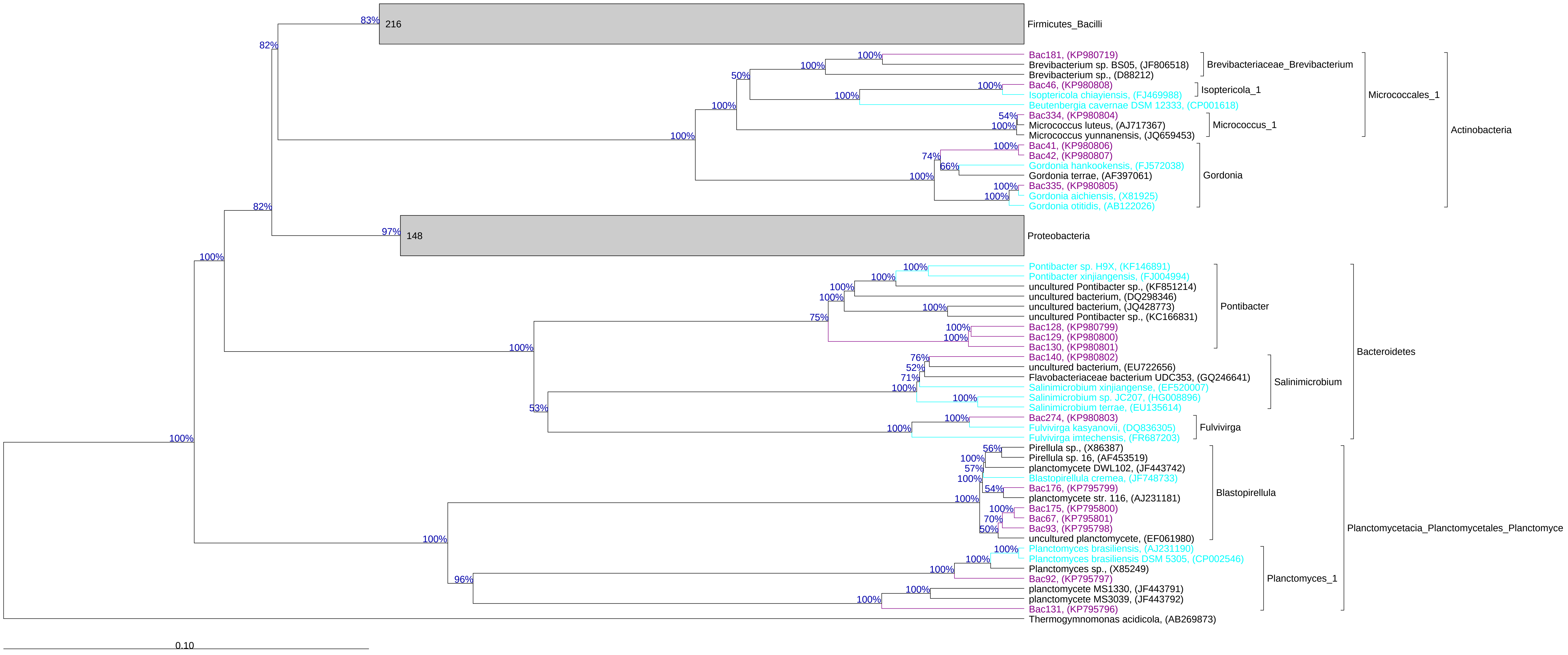

Figure S1. Phylogenetic tree showing the relationship of the all cultured bacterial strains. The sequence alignment was performed using the SINA online tool (http://www.arb-silva.de/aligner) and trees were built in ARB starting from the SILVA SSU dataset Ref NR 99 (version 119) using MrBayes with 100,000 Markov Chain Monte Carlo (mcmc) cycles.
